# Technology Management as a Core Component of a Client-centric Prosthetic Orthotic Practice Model

**DOI:** 10.33137/cpoj.v5i2.39001

**Published:** 2022-12-31

**Authors:** S.U. Raschke

**Affiliations:** British Columbia Institute of Technology (BCIT), 3700 Willingdon Avenue, Burnaby, British Columbia, Canada.

**Keywords:** Orthosis, Prosthesis, 3D Printing, Prosthetics, Orthotics, Funding, Economic, Health Care, Assistive Technology

## Abstract

Technological innovation has transformed how we communicate, work, and conduct business. Over the next decade how we experience health care both as health care professionals and as client-patients will also change significantly. This presents both an opportunity and a challenge to medical clinical professionals that are device-focused, including prosthetists orthotists, as they consider how best to adapt. Current prosthetic orthotic education and practice is heavily clinically weighted, with less emphasis being given to engineering and business skills. Yet all three are essential core elements of a successful, sustainable prosthetics orthotics practice. Furthermore, it is the latter two that will heavily influence the future face of prosthetics & orthotics. It is not certain how current prosthetic orthotic practitioners can best adapt in response. One solution, proposed in this editorial, could be by rebalancing their professional persona to equally weight the three essential core elements. The result, a Clinical Prosthetic Orthotic Technology Management Professional, would engage in a professional practice that is functionally grounded, uses a client-centric model and incorporate eight professional attributes: professional, advocate, scholar, leader, communicator, collaborator, assistive technology expert and business justification specialist.

Technological innovation has transformed how we communicate, work and conduct business over the past two decades and is now visibly evolving the health care sector. Technology translating into health includes: data collection tools, smart technology, new communication platforms and additive manufacturing, with the objectives of improving access to care, outcomes and productivity. The new technology also potentially supports the concept of personalized medicine and creating opportunities for patients to becoming actively engaged in health care decisions as a client-patient. Over the next decade, additional tools will be developed that will significantly change how we experience health care both as a health care professional and as the client-patient.

Being device oriented, prosthetics and orthotics (P&O) examples are commonly cited as what transformative technology in health looks like, with examples ranging from exoskeletons, to microprocessor-controlled components, to sensors and the ever-popular 3D printing (additive manufacturing) of prosthetic and orthotic devices. All consistently capture the attention of the media and imagination of the public.^1^

A range of the hands-on activities once done manually by the prosthetist orthotist are becoming digitalized and data driven through the use of practice management software, clinical outcome measures, scanners, digitizers and remote fabrication options. In parallel, the palette of componentry and device deigns available to address a client-patient's needs are expanding rapidly to include highly sophisticated and complex components, such as sensors giving sensory feedback, alongside simpler approaches such as comparatively low cost and easy to fit supportive smart apparel that replaces a number of hard orthotic brace designs.

These changes present both an opportunity and a challenge to prosthetists orthotists. Extensive effort was made to update prosthetic orthotic education over the past two decades in many countries globally. The changing curriculum typically emphasised the development of a solid, deeper and broader understanding of the clinical aspects of prosthetics orthotics. The same emphasis was not given to broadening and deepening the knowledge base supporting prosthetic orthotic design and engineering principles or on the critical economic and business complexities which govern the prosthetics orthotics market. In hindsight, this overemphasis on one of what are three core elements that are required for the successful and sustainable practice of prosthetics orthotics, has made the sector vulnerable to disruption by external forces with skills and expertise in the two underemphasized elements.

The dilemma presented by this imbalance became visible to the BCIT MAKE+ applied research group as early as 2015. Having been using 3D printers in medical and assistive device prototyping since 2001 we saw a sharp increase of potential clients seeking to engage us to apply 3D printing to the production of orthotic devices, citing known challenges with the current provider model and untapped market potential. They had identified prosthetics orthotics as a sector ready for disruption.

To explore the opportunity further, a workshop was held inviting a small, international group of engineering and clinical prosthetic orthotic academics, alongside end user representatives, to grapple with how emerging technologies could impact current device provision processes and business models using a SWOT (Strength, Weakness, Opportunity, Threat) model. It was during this day that one of our facilitators commented: “*It seems to me that your problem is one of technology management*”.

The ensuing discussion stayed with me. He did not mean that emerging tools and technologies were a problem to be managed. What he meant was that prosthetist orthotist's ability to adopt and mange new technology was key to the sector surviving and thriving. A clear threat identified that day was that technology innovation well beyond 3D printing was allowing non-traditional actors to insert themselves into the provision process by creating potentially better designs at lower price points – a legitimately attractive value proposition to payors.

His pointed out that the traditional value proposition of the prosthetist orthotist has been that they are the best, or most knowledgeable person, to accompany the client-patient on the path from prescription to receiving a functioning and well-fitting device. This was a reasonable assumption at a time, when the device production process required specialized skills and equipment along with access to prosthetic orthotic components from component manufacturers who only sold them to recognized prosthetists orthotists. The result was an ecosystem that was easy to control, as long as payors continued to accept that value proposition. Unfortunately, the proposition was vulnerable at two of the three core elements identified above; vulnerabilities which had been identified as opportunities by the tech development community. Unless prosthetists orthotists begin to develop strengths in those two underemphasized core elements, maintaining the current model will become challenging.

The next evolution of health care is being carried out by well funded teams of technical experts working with tech sector business strategists and is attracting the attention of large companies and investment funds that previously had not been active in the medical device sector.^2,3^ Device-based and fee-for-device elements of the health care system have been identified as ideally situated for positive disruption. Engineers and industrial designers have become recognized as partners in improving health care delivery as technology-based solutions begin to permeate every aspect of health care under what has been identified as Medicine 4.0.^4^ It is difficult for small professions to keep pace with such co-ordinated and well funded impetus, to say little of maintaining control and attempting to guide it.

Given the unavoidability of this newly developing ecosystem, the facilitator suggested that there is the potential for prosthetist orthotist to secure their place proactively by proposing a new value proposition and repositioning themselves as “*technology managers”*. This repositioning would see the role of the prosthetist orthotist broaden slightly from a medical-clinical focus to one that also guides client-patients in navigating the increasing number of technology options, which will come in a range of price points, to arrive at optimized and affordable solution for a client-patient. The final solution arrive at might take the form of a custom made and fitted device or might not.

Such a repositioning would not require abandoning the current clinical role of the prosthetist orthotist. Instead, it would be a re-balancing of their professional identity to include, with equal emphasis, all three core elements of the prosthetic orthotic provision model identified above. It would also reduce the fear associated with new technologies disrupting the current provision model and dampen frustrations linked to the reimbursement process. Strengthening competencies in the two weak core elements will allow prosthetist orthotists to master those core elements, as opposed to being controlled by them.

Such a shift is not outside the realm of possibility, with a number of sources already pointing in directions compatible with such a rebalancing. Without being explicitly stated as part of a Client-centric Care Model, technology management is already, informally, an ad hoc part of the device provision process. In a recent professional opinion written by Dr. Chris Hovorka (2022) it is proposed that the next generation of prosthetic orthotic education will be based on a curriculum that is functionally focused, as opposed to disease or condition focused, using the World Health Organization's (WHO) International Classification of Functioning, Disability and Health (ICF) model.^5^ He suggests informing such a framework with the Prosthetic and Orthotic Practice (POP) model described by Jahn and Ramsted,^6^ who describe a model for how ICF can be applied within prosthetic orthotic curriculum and practice. Finally, he suggests adopting the six key attributes from the competency based medical education framework developed by the Royal College of Physicians and Surgeons of Canada as part of their Competence by Design initiative (CanMEDS)^7^ into the prosthetic orthotic professional persona. These are: professional, advocate, scholar, leader, communicator and collaborator, and have been identified as components necessary for physicians to serve their client-patients well. Considering the threats and stressors to the current prosthetics orthotics practice models identified in the 2018 BCIT workshop, it could be proposed that two further attributes specific to the prosthetic provision process should be added, namely, assistive technology expert and business justification specialist.

An amalgam of the above would create a new practice model that could be described as a Clinical Prosthetic Orthotic Technology Management Professional, encompassing all three core elements of a successful and sustainable prosthetic orthotic provision process with equal weight and competency. As a Client-centric model it recognizes what prosthetists orthotists already do, strengthens the two weak core practice elements that currently leave the sector vulnerable to disruption and does so using an inclusive model which engages the client-patient in decision making and cocreation processes.^8^ It remains to be seen what the future will bring, but what is already clear is that all device-based health services will require a high level of technology management activities, whether officially recognized and supported or not.

## DECLARATION OF CONFLICTING INTERESTS

Dr. Silvia Raschke is a member of the Midwestern University's Advisory Board for the Prosthetics and Orthotics M.Sc. Program as of Spring 2022 and in this capacity has participated in discussions that may have influenced her opinions on this topic. These opinions and ideas expressed in the editorial are those of Dr. Raschke and have not been reviewed by or endorsed by Midwestern University.

## SOURCES OF SUPPORT

None.

## References

[1] Birrill I. 3D-printed prosthetic limbs: the next revolution in medicine [Internet]. The Guardian, 2017; [cited 2022 July 5] Available from: https://www.theguardian.com/technology/2017/feb/19/3d-printed-prosthetic-limbs-revolution-in-medicine

[2] Parsons C. Data, data everywhere: why the medical device industry must embrace the fourth industrial revolution [Internet]. Medical Design Briefs, 2018; [cited 2022, July 5]. Available from: https://www.medicaldesignbriefs.com/component/content/article/mdb/features/articles/29115

[3] Saunders S. Revenue from 3D printed prosthetics, orthotics, & audiology to reach nearly $1B by 2030, says SmarTech Analysis [Internet]. 3Dprint.com, 2021; [cited 2022, July 5], Available from: https://3dprint.com/283008/revenue-from-3d-printed-prosthetics-orthotics-audiology-to-reach-nearly-1b-by-2030-says-smartech-analysis/

[4] Popov VV, Kudryavtseva EV, Kumar Katiyar N, Shishkin A, Stepanov SI, Goel S. Industry 4.0 and digitalisation in healthcare. Materials. 2022; 14;15(6):2140. DOI: 10.3390/ma15062140 PMC8953130 35329592

[5] Hovorka, C. O&P education: guiding the transition to client-centric training [Internet]. The O&P Edge, 2022; [cited 2022, July 5], Available from: https://opedge.com/op-education-guiding-the-transition-to-client-centric-training/

[6] Jarl G, Ramstrand N. a model to facilitate implementation of the international classification of functioning, disability and health into prosthetics and orthotics. Prosthet Orthot Int. 2018;42(5):468-475. DOI: 10.1177/0309364617729925 28905670 PMC6146308

[7] Frank JR, Snell L, Sherbino J, editors. CanMEDS 2015-physician competency framework. Ottawa: Royal College of Physicians and Surgeons of Canada; 2015. ISBN: 978-1-926588-28-5

[8] Desmond D, Layton N, Bentley J, Boot FH, Borg J, Dhungana BM, et al. Assistive technology and people: a position paper from the first global research, innovation and education on assistive technology (GREAT) summit. Disabil Rehabil: Assist Technol. 2018;13:5,437-444.DOI: 10.1080/17483107.2018.1471169 29772940

